# Dietary patterns are associated with improved ovarian reserve in overweight and obese women: a cross-sectional study of the Lifestyle and Ovarian Reserve (LORe) cohort

**DOI:** 10.1186/s12958-022-00907-4

**Published:** 2022-02-19

**Authors:** Ashley M. Eskew, Bronwyn S. Bedrick, Jorge E. Chavarro, Joan K. Riley, Emily S. Jungheim

**Affiliations:** 1 Department of Obstetrics and Gynecology, Washington University School of Medicine, St. Louis, MO 63110 USA; 2 Present address: Department of Obstetrics and Gynecology, Atrium Health, Charlotte, NC 28204 USA; 3 Present Address: Department of Obstetrics and Gynecology, Johns Hopkins University, Baltimore, MD 21287 USA; 4 Harvard T. H. Chan School of Public Health, Harvard University, Boston, MA 02115 USA; 5 Present address: Department of Obstetrics and Gynecology, Northwestern University Feinberg School of Medicine, Chicago, IL 60611 USA

**Keywords:** Profertility diet, Ovarian reserve, Fertility, Obesity, Fertility diet

## Abstract

**Background:**

Growing evidence suggests that adherence to certain dietary patterns is associated with improved fecundity and reproductive outcomes in the general population and infertile couples assisted reproductive treatments. The objective of this study was to assess if dietary patterns are associated with ovarian reserve in reproductive age women without a history of infertility.

**Methods:**

This was a cross-sectional study of 185 women in the Lifestyle and Ovarian Reserve (LORe) cohort. Women aged 18–44 without a history of infertility were recruited from the local community at an academic medical center. Subjects completed validated food frequency and physical activity questionnaires to assess patterns over the year prior to presentation. Dietary patterns including a Western (including meat, refined carbohydrates, high-calorie drinks), prudent (including fruits, vegetables, olive oil and nuts), fertility (lower intake of *trans* fat with higher intake of monounsaturated fatty acids, increased intake of plant based protein, high-fat dairy, lower glycemic load carbohydrates and supplemental iron) and profertility diet (PFD) (characterize by whole grains, soy and seafood, low pesticide residue produce, supplemental folic acid, B12 and vitamin D) were identified through principal component analysis. Main outcome measures were serum antimullerian hormone concentration (AMH) (ng/mL) and antral follicle count (AFC) obtained by transvaginal ultrasound.

**Results:**

After stratifying by BMI, adjusting for age, smoking and physical activity, dietary patterns were not associated with ovarian reserve in normal weight women. Increased adherence to a profertility diet in overweight and obese women (BMI ≥ 25 kg/m^2^) was associated with a significantly higher AMH. Women in the third and fourth quartiles of PFD adherence had a mean AMH concentration of 1.45 ng/mL (95%CI 0.33–2.56, *p* = 0.01) and 1.67 ng/mL (95%CI 0.60–2.74, *p* = 0.003) higher than women in the lowest quartile respectively. The highest adherence to PFD was also associated with a higher AFC in women with a BMI ≥ 25 kg/m^2^ (β = 7.8, 95%CI 0.003–15.34, *p* < 0.05). Other common dietary patterns were not significantly associated with ovarian reserve.

**Conclusions:**

Increased adherence to a profertility diet is associated with improved markers of ovarian reserve in overweight and obese women. These findings provide novel insight on potential modifiable lifestyle factors associated with ovarian reserve.

## Background

Studies have shown that dietary patterns are associated with endometriosis, ovulatory infertility and fecundability [1–5]. Additionally, emerging evidence suggests that modifiable lifestyle factors, including adherence to certain dietary patterns and avoidance of environmental toxins, are associated with improved reproductive outcomes, including clinical pregnancy, spontaneous miscarriage and live birth rates, in women undergoing in vitro fertilization (IVF) [6, 7].

The Fertility Diet (FD) was first described in 2007 from the Nurses’ Health Study II [5]. This diet, which is characterized by lower intake of *trans* fat and higher intake of monounsaturated fatty acids, plant-based protein, and high-fat dairy, has been shown to associated with the lowest risk of ovulatory infertility. In 2019, pretreatment adherence to the Profertility Diet (PFD) was found to be associated with an increased probability of live birth in women undergoing in vitro fertilization and embryo transfer when compared to other dietary patterns. The PFD is characterized by increased intake of whole grains, soy and seafood (in place of conventional animal protein), dairy, low pesticide residue produce [6]. Despite increasing data on the association of lifestyle and dietary patterns and reproductive outcomes, evidence regarding the influence of diet on measures of ovarian reserve is scarce [8, 9]. Markers of ovarian reserve, including anti-Müllerian (AMH) hormone concentration and antral follicle count (AFC) are used to predict ovarian responsiveness to gonadotropins in women undergoing ovarian stimulation [10–12].

Women with overweight and obesity have been shown to have decreased ovarian responsiveness to oral ovulation induction medications and gonadotropin stimulation [13–15]. Further AMH has been shown to be inversely associated with increasing body mass index (BMI) [16]. Being able to provide evidence-based lifestyle recommendations to optimize outcomes in reproductive age women seeking fertility treatments is critical. Given evidence that adherence to certain dietary patterns is associated with reproductive outcomes we hypothesized that dietary patterns are also associated with markers of ovarian reserve as determined by AFC and AMH concentration. We sought to investigate this in a novel cohort of reproductive age women without a history of infertility with normal weight, overweight or obesity.

## Methods

For this cross-sectional study, reproductive age women were recruited from the St. Louis, Missouri metropolitan area for participation in the LORe study—lifestyle and reproductive outcomes—between May 2014 and May 2018 [17]. Briefly, women 18 to 44 years of age with regular menstrual cycles were eligible for the study. Exclusion criteria included history of ovarian surgery or infertility, PCOS as defined by NIH criteria, a major chronic disease (e.g., diabetes mellitus, hypertension, autoimmune disease), or current pregnancy.

A sample size of 200 women was initially calculated assessing rates of diminished ovarian reserve (as measured by AFC) between women with low or normal omega-6:omega-3 ratios assuming a 0.05 type I error, and 0.20 type II error and were enrolled in this study based on findings from prior work [18]. This study was approved by the Institutional Review Board at Washington University School of Medicine (#201405045). Subjects were enrolled after obtaining informed consent. The analysis of this cross-sectional study was performed according to STROBE guidelines [19].

Participants completed three questionnaires: a general demographic questionnaire, the Harvard Willett Food Frequency Questionnaire, and the Kaiser Physical Activity Survey [20–22]. The general questionnaire contained questions on race, ethnicity, obstetric history, past and present use of contraceptives, and past and present smoking status.

The Harvard Willett Food Frequency Questionnaire was used to capture participants’ diet over the previous year [21, 22]. The questionnaire asked participants to report how often, on average, they consumed a specified amount of 131 foods, beverages, and supplements. Quantifications of food items were based on commonly used units (e.g. one banana, 8 oz. skim milk). Individual food items were then combined to form 40 predefined food groups (e.g., low-fat dairy products) based on nutrient profile and culinary use, as previously described [23]. Daily consumption of these food groups was then calculated. From these quantifications, adherence to different diets was calculated. Women with caloric intake < 500 kcal/day or > 5000 kcal/day were excluded from analyses (*n* = 9).

The Kaiser Physical Activity Survey (KPAS) [20] was used to capture participants’ physical activity over the previous year. In brief, KPAS consists of four indices: household and caregiving; occupational; active-living; and sports and exercise. The occupational index was modified to exclude the occupational intensity component, as previously described [24, 25] and individuals who were unemployed were assigned a value of one. To calculate the sports and exercise index, metabolic equivalent ratings from the Compendium of Physical Activity were assigned to each listed activity [26].

Transvaginal ultrasound was performed to determine the total AFC, which was calculated as the sum of antral follicles measuring 2-10 mm in both ovaries [27]. Serum AMH concentration was determined using a Roche cobas e411 analyzer. BMI was calculated from the height and weight measured at each participant’s research visit.

Adherence to predefined dietary patterns and the common dietary patterns in our cohort were calculated from each participant’s daily food consumption. For the FD and PFD, points were assigned to food groups most associated with fertility, as described in Chavarro et al. and Gaskins et al., respectively [5, 6]. For the PFD, pesticide residue burden was determined by pesticide residual based scoring which did not account for intake of organic produce [28].

In addition to analyzing the predefined FD and PFD, we used principal components analysis to identify and categorize the underlying dietary patterns among study participants [29–31]. Varimax rotation was used for greater interpretability. Principal components analysis allowed us to synthesize the extensive dietary data from the Harvard Food Frequency Questionnaire into a smaller number of variables—or dietary patterns. Given significant regional variability in diet [32, 33], using principal components analysis permitted us to explore dietary patterns unique to our cohort. The number of dietary patterns retained was determined according to three considerations: eigenvalues > 1.0, scree plot, and pattern interpretability. Food groups were considered discriminatory if their loading factors were ≥ |0.4|. The dietary pattern score for each woman was determined by adding her daily consumption of each food group multiplied by the standardized coefficients of each food group. Women with higher scores for a specific dietary pattern were considered to be more adherent to that dietary pattern which has been described previously [17].

To assess the association between markers of ovarian reserve and each dietary pattern, multivariate linear regression was then performed controlling for age, BMI, smoking status, and physical activity. Subjects were stratified by BMI < 25 kg/m^2^ and BMI ≥ 25 kg/m^2^ as BMI is known to be associated with markers of ovarian reserve [16]. KPAS and the dietary pattern scores were divided into quartiles, with the lowest quartile representing women least adherent to the behavior. Variables included in the final model were based on univariate analysis and known confounders based on prior literature [34–36]. Spearman correlation was used to assess strength of correlation between the different dietary patterns.

All data were stored on the online database REDCap [37]. *P* < 0.05 was considered statistically significant. SAS version 9.4 (SAS Institute, Cary, NC, USA) was used for all statistical analyses.

## Results

A total of 185 women were recruited and 175 were included in the analysis. Women were excluded from analysis if they did not complete all aspects of the study (*n* = 1) or if they had a daily caloric intake > 5000 or < 500 kcal per day (*n* = 9). Women who had AFC documented from only one ovary were included for analysis of AMH but not AFC (*n* = 8). Participants had a mean age of 31.0 (±6.6) years and had a mean BMI of 27.7 (±7.0) kg/m^2^. The majority were non-smokers (92%), Caucasian (61%) and had graduated from college (71%).

For the overall cohort, the median (25th–75th percentile) AMH concentration was 2.2 (1.3–3.6), and the median AFC was 25 (17–37). For women with a BMI ≥25 kg/m^2^, the median AMH was 2.2 (1.3–2.6) and the median AFC was 24 (15–31). For women with a BMI < 25 kg/m^2^, the median AMH was 4.5 (3.0–5.8) and AFC was 27 (19–41).

We identified two dietary patterns by principal components analysis. The first pattern was characterized by high intake of animal protein, snack foods, potatoes, and high-calorie drinks. We termed this dietary pattern the “Western” pattern. The second pattern, which we termed the prudent diet, was characterized by increased consumption of fruits, vegetables, and nuts**.** These patterns’ characterizations were similar to those described before in the literature, however, regional differences were noted between the prudent dietary pattern in our cohort and that which has been described in the literature. For example, the prudent diet in our cohort was not defined by consumption of fish [17]. These dietary patterns were then compared to subjects with increased adherence to the PFD and FD.

The median (25th–75th percentile) score of the PFD was 18 (16–21) overall. For women with a BMI ≥ 25 kg/m^2^, it was 18 (16–21) and 18 (15.5–21) for women with BMI < 25 kg/m^2^. The median score for the FD was 19 (16–21) overall. For women with BMI ≥25 kg/m^2^ the median score was 18 (16–21) and 19 (18–21) for women with BMI < 25 kg/m^2^. There was a moderate positive correlation between the PFD and FD (rho = 0.19, *p* = 0.01), and a strong positive correlation between the PFD and the Prudent diet (rho = 0.37, *p* < 0.0001). The FD and Western diet had a strong inverse correlation (rho = − 0.56, p < 0.0001).

Women with increased adherence to the PFD were more likely to be older, college educated, have higher physical activity levels, greater daily caloric intake, consume higher amounts of alcohol and caffeine, and were more likely to take a daily multivitamin than women with decreased adherence [Table 1]. Women with increased adherence to the FD were more likely to have higher physical activity levels, higher educational attainment, to have lower BMI, to have lower daily calorie intake, and to have higher multivitamin intake (data not shown). Women with increased adherence to the Prudent diet were also more likely to be college educated, to have higher physical activity levels, and to have lower BMIs. Women with increased adherence to the Western diet were more likely to be older, to have lower educational attainment, and to have higher BMI.

**Table 1:** Baseline characteristics by adherence to the Profertility Diet

Characteristics	Profertility diet				
Quartile	Quartile 1 (***n*** = 41)	Quartile 2 (***n*** = 55)	Quartile 3 (***n*** = 40)	Quartile 4 (***n*** = 39)	***P***-value
BMI < 25 kg/m^2^ (n)	19	23	18	16	
BMI ≥ 25 kg/m^2^ (n)	22	32	22	23	
Age (years)	27 (24–32)	31 (25–37)	31 (26–36)	31 (27–38)	0.06
Current smoker	4 (10%)	4 (7.4%)	2 (5%)	4 (10.3%)	0.85
White	23 (58%)	29 (53%)	27 (69%)	28 (72%)	0.19
Education					0.08
Less than college	15 (38%)	20 (36%)	10 (26%)	6 (15%)	
College graduate or higher	25 (62%)	35 (64%)	29 (74%)	33 (85%)	
BMI (kg/m^2^)	26.2 (23.1–33.3)	25.8 (22.8–32.9)	26.7 (22.9–30.0)	25.7 (22.3–29.9)	0.82
KPAS	9.3 (8.1–11.1)	9.5 (8.2–11.4)	11.1 (10.3–11.6)	10.4 (9.0–11.9)	0.002^a^
AMH	2.6 (1.8–4.1)	2.3 (1.3–3.8)	2.7 (1.6–4.3)	2.8 (1.7–4.5)	0.85
AFC	25 (12–38)	22 (15–31)	24 (17–38)	28 (17–35)	0.71
Dietary Characteristics					
Total calories, kcal/d	1500 (1112–1747)	1705 (1270–2045)	2054 (1603–2592)	2013 (1761–2430)	< 0.0001
Carbohydrates, % of kcal/d	48 (42–53)	47 (40–54)	48 (43–50)	46 (42–51)	0.91
Protein, % of kcal/d	17 (14–19)	17 (13–19)	17 (15–18)	17 (16–19)	0.28
Fat, % of kcal/d	34 (30–44)	34 (30–40)	34 (32–37)	34 (31–38)	> 0.999
Alcohol, g/d	2.3 (0–6.6)	3.6 (0–8.1)	5.6 (2.0–10.3)	7.0 (2.5–11.8)	0.01
Caffeine, mg/d	31.6 (13.1–114.7)	95.2 (16.0–239.0)	115.8 (45.8–237.2)	161.5 (91.5–288.5)	0.0001
Multivitamin use	6 (15%)	14 (26%)	18 (47%)	29 (74%)	< 0.0001

Note: Categorical data are represented as n (%), and continuous variables are represented as median (interquartile range)

Note: *KPAS* Kaiser physical activity survey, *AMH* Anti-Müllerian, *AFC* antral follicle count

^a^*P* < 0.05 across groups

No association was observed between PFD adherence and ovarian reserve in our overall cohort after adjusting for age, smoking status and physical activity (data not shown). However, after stratifying by BMI, adherence to the PFD in overweight and obese women (BMI ≥ 25 kg/m^2^) was associated with higher AMH concentrations. Women in the third and fourth quartiles of the PFD had mean AMH concentrations of 1.45 ng/mL (95%CI 0.33–2.56, *p* = 0.01) and 1.67 ng/mL (95%CI 0.60–2.74, *p* = 0.003) higher than women in the lowest quartile respectively. The highest adherence with the PFD was also associated with a higher mean AFC in overweight and obese women (β = 7.8, 95%CI 0.003–15.34, *p* = 0.049) whereas quartiles 2 and 3 were not. Increased adherence to PFD not associated with markers of ovarian reserve in normal weight women [Table 2].

**Table 2:** Association Between Adherence to Dietary Patterns and Markers of Ovarian Reserve by BMI

Variables	AMH, (95%CI)	*P* value	AFC, (95%CI)	*p*-value
**Profertility Diet**				
BMI < 25				
Q1 (reference)	–		–	
Q2	−0.78 (−1.97–0.40)	0.19	−3.46 (− 11.9–5.01)	0.42
Q3	− 0.31 (− 1.64–1.02)	0.65	7.17 (−2.47–16.8)	0.14
Q4	− 0.87 (− 2.20–0.46)	0.20	0.19 (− 9.24–9.61)	0.97
BMI ≥ 25				
Q1 (reference)	–		–	
Q2	0.86 (−0.12–1.84)	0.08	5.9 (−1.2–13)	0.1
Q3	1.45 (0.33–2.56) ^b^	0.01	5.93 (−2.41–14.3)	0.16
Q4	1.67 (0.60–2.74) ^b^	0.002	7.77 (0.004–15.4) ^b^	0.049 ^b^
**Fertility Diet**				
BMI < 25				
Q1 (reference)	–			
Q2	−0.47 (−1.93–0.99)	0.52	−4.16 (− 15.49–7.17)	0.47
Q3	0.07 (−1.22–1.37)	0.91	0.41 (−9.00–9.81)	0.93
Q4	0.39 (−0.98–1.75)	0.57	−1.16 (− 11.11–8.78)	0.82
BMI ≥ 25				
Q1 (reference)	–			
Q2	0.74 (−0.26–1.74)	0.14	1.04 (−6.1–8.19)	0.77
Q3	0.77 (−0.51–2.07)	0.23	0.92 (−8.29–10.14)	0.84
Q4	0.84 (−0.11–1.79)	0.08	−0.28 (−7.16–6.61)	0.94
**Traditional Diet**				
BMI < 25				
Q1 (reference)	–		–	
Q2	0.34 (−0.71–1.39)	0.52	1.33 (−6.74–9.40)	0.74
Q3	0.63 (−0.55–1.80)	0.29	0.24 (−8.66–9.15)	0.96
Q4	−0.97 (−2.25–0.31)	0.13	−2.61 (−12.23–7.01)	0.59
BMI ≥ 25				
Q1 (reference)				
Q2	−0.15 (−1.35–1.04)	0.80	1.70 (−6.57–9.98)	0.68
Q3	0.33 (−0.79–1.45)	0.56	5.13 (−2.71–12.98)	0.20
Q4	−0.16 (−1.31–0.99)	0.78	− 0.17 (−8.21–7.86)	0.97
**Prudent Diet**				
BMI < 25				
Q1 (reference)	–		–	
Q2	−0.71 (−2.18–0.77)	0.34	−7.09 (−17.50–3.31)	0.18
Q3	−0.32 (−1.50–0.86)	0.59	1.90 (−6.66–10.47)	0.66
Q4	0.09 (−1.27–1.44)	0.90	−8.65 (− 18.42–1.12)	0.08
BMI ≥ 25				
Q1 (reference)				
Q2	0.47 (−0.53–1.47)	0.35	−1.25 (−8.31–5.81)	0.73
Q3	0.62 (−0.63–1.87)	0.33	3.85 (−4.91–12.59)	0.38
Q4	0.55 (0.60–1.66)	0.33	1.16 (−6.64–8.97)	0.77

Note: *CI* Confidence interval, *Q* Quartile

^a^Analyses were run using multivariate linear regression. Data are presented as the difference in means (95% CI) adjusted for age, BMI, smoking status and physical activity level

^b^*P* < 0.05 for comparison of designated quartile to quartile 1 (reference)

The fertility diet, prudent diet and Western dietary patterns were not significantly associated with AMH or AFC in normal weight, overweight or obese women [Table 2].

## Discussion

In this cross-sectional cohort study of reproductive age women without a history of infertility, we observe a linear association with improved markers of ovarian reserve in women with overweight and obesity with increasing adherence to the PFD. This association remained after controlling for potential confounders. Markers of ovarian reserve were negatively associated with increasing adherence to a Western dietary pattern, but this did not reach statistical significance at any level of adherence. Increased adherence to the fertility diet was not associated with markers of ovarian reserve in women with normal, overweight or obese BMIs.

Increasing evidence over the past decade has shown that lifestyle factors including diet and exercise patterns are associated with fecundability in a general population of reproductive age women trying to conceive [1–3, 38]. Our study is one of the first to investigate associations between dietary patterns and markers of ovarian reserve in a reproductive age cohort of ovulatory women without a history of infertility.

Anderson et al. evaluated the association between dietary intake and serum AMH concentrations among 296 late premenopausal women in the Sister Study. Their findings suggest that dietary fat intake may be inversely associated with AMH concentration. However, their cohort had a mean age of 42.8 years, a median BMI of 25 kg/m^2^ and no specific dietary patterns were examined for associations [39]. The EARTH study, a study of women undergoing in vitro fertilization, demonstrated no relation of dietary patterns to AFC but did suggest an inverse relation of AFC to dairy intake specifically [8, 9]. They did not look at AMH, a more objective and reliable measure of ovarian reserve.

Data from the Nurses’ Health Study II (NHSII), which served as the foundation for the fertility diet, examined 18,555 married, premenopausal women without a history of infertility who had attempted pregnancy during an eight-year period during the study. Dietary assessments were made in relation to the incidence of ovulatory infertility. During the follow up period, women who consumed higher amounts of *trans* fats as opposed to monounsaturated or polyunsaturated fats had a significantly higher risk of ovulatory infertility [40]. Additionally, women who consumed the highest glycemic load carbohydrates compared to the lowest amounts, had a 92% higher risk of ovulatory infertility, whereas women who prioritized intake of plant based proteins and took a daily multivitamin conversely had a 22 and 41% lower risk of ovulatory infertility respectively [41, 42]. Our cohort differs from the NHSII in that women with PCOS were specifically excluded from our study. One factor that wasn’t accounted for by the Nurse’s Health Study II was intake of low versus high pesticide residue fruits and vegetables which is important given the evidence regarding the impact of environmental toxins (including through our food systems) on reproductive health [43].

Increased intake of high pesticide residue fruits and vegetables has been directly associated with increased systemic levels of pesticide metabolites [44]. In women undergoing IVF, increased consumption of high pesticide fruits and vegetables (> 2.3 servings/day versus < 1.0 servings per day) was associated with an 18% lower probability of clinical pregnancy and 26% lower probability of live birth [44]. When combining these findings with assessment of dietary patterns the association with reproductive outcomes has been substantiated [6].

Gaskins et al. was the first to describe a “profertility” diet that was characterized by higher intake of low pesticide residue fruits and vegetables, whole grains, seafood, dairy, soy foods and supplemental vitamin D, folic acid and B12. Women undergoing IVF with the highest adherence to a PFD had significantly higher odds of implantation (47%), clinical pregnancy (43%) and live birth (53%), and a 31% lower odds of clinical pregnancy loss. Higher adherence to the fertility diet, in contrast, was not associated with reproductive outcomes following IVF. These findings highlight the importance of accounting for environmental exposures such as pesticide residue burden when examining lifestyle factors that may impact reproductive health and outcomes as our study does here.

In addition to modifiable lifestyle factors, obesity is associated with a longer time to pregnancy, higher likelihood of menstrual cycle irregularity [45, 46], ovulatory dysfunction [47, 48] and decreased ovarian responsiveness to oral ovulation induction medications and gonadotropin stimulation [13–15]. BMI at age 18 has been shown to be a predictor of future ovulatory infertility [47]. Further, studies have demonstrated that BMI has an inverse association with AMH, making the findings we present here even more compelling [16]. Our findings are important as they offer the potential for building evidence-based tools tailored to help empower at-risk population for improved fertility outcomes.

Our results may also be beneficial for women undergoing ovarian stimulation for planned oocyte cryopreservation or other clinical indications. AMH concentration and AFC have been shown to correlate with ovarian responsiveness to gonadotropins and to be accurate predictors of poor response [10, 12, 36]. Implementation of a PFD pattern as discussed could further optimize assisted reproductive treatment outcomes particularly in patients with a poor prognosis such as those with diminished ovarian reserve.

Lastly, AMH concentration has been shown to potentially predict age of menopause, with lower AMH concentration being associated with earlier onset [11]. Early age of menopause has been shown to be associated with a higher risk of bone, neurologic and cardiovascular disease in women [49–52]. Simple dietary counseling could be implemented as preventative management in reproductive age women to potentially lessen their risk of early menopause and associated co-morbidities.

There are many strengths of this study including its novel findings. This was also a reproductive age cohort of women without a history of chronic disease which could minimize additional confounders. Additionally, we used principal components analysis to identify dietary patterns present in our cohort and examined pre-defined dietary patterns. This permitted us to determine if dietary patterns other than those that have been previously described could explain differences in markers of ovarian reserve. Using dietary patterns rather than looking at dietary intake or serum concentrations of specific nutrients allows for improved tools for counseling women about modifiable lifestyle factors and reproductive health. Further, the use of KPAS more accurately assesses physical activity in women compared to other questionnaires which allowed for better assessment of a potential confounder.

Several limitations must be considered in the interpretation of our work. First, our sample size was small. While sample size may impact accuracy, our findings were significant and based on our a priori hypothesis, and therefore these findings are worthy of consideration and future prospective investigation. Our cohort was comprised of healthy, reproductive age women from a mid-west metropolitan area. This may limit generalizability to women in other regions and certain dietary patterns associated with chronic disease. Additionally, it’s being increasingly recognized that the US lags behind other countries in limiting the use of harmful pesticides, so these findings may not be generalizable world-wide [53]. Although our dietary questionnaire did not account for organic produce intake future studies should account for this. While we demonstrated an association between dietary patterns and AMH concentration in women with overweight or obesity, these findings were not shown in women with normal weights. As ovarian reserve decreases throughout a woman’s reproductive years, it is difficult to determine the most relevant biologic window to assess dietary patterns and measures of ovarian reserve. Furthermore, our cross-sectional study design limits our ability to determine causality, although based on other prospective cohort studies of diet and reproductive health, our results are biologically plausible.

## Conclusions

Findings from this cross-sectional study of reproductive age women without a history of infertility support the association between dietary patterns and markers of ovarian reserve, but only in overweight and obese women. Additional prospective studies are needed to evaluate the potential modifying effect of dietary intake on ovarian reserve.

## Data Availability

Data cannot be shared for ethical/privacy reasons. The data underlying this article cannot be shared publicly for the privacy of individuals that participated in the study. The data will be shared on reasonable request to the corresponding author.

## Abbreviations

AFC: Antral follicle count
AMH: Antimullerian hormone concentration
BMI: Body mass index
FD: Fertility Diet
IVF: In vitro fertilization
KPAS: Kaiser Physical Activity Survey
LORe: Lifestyle and Ovarian Reserve
PFD: Profertility Diet
